# Editorial: Roles and regulatory mechanisms of ABA in plant development

**DOI:** 10.3389/fpls.2022.1039265

**Published:** 2022-10-20

**Authors:** Guanghao Li, Yifeng Wang, Jian Wu, Honghong Hu, Raju Datla, Qingyun Bu, Paloma Moncaleán, Jiaxu Li, Jian Zhang

**Affiliations:** ^1^ State Key Lab of Rice Biology, China National Rice Research Institute, Hangzhou, China; ^2^ Beijing Key Laboratory of Development and Quality Control of Ornamental Crops, Department of Ornamental Horticulture, China Agricultural University, Beijing, China; ^3^ Key Laboratory of Crop Genetic Improvement, Hubei Hongshan Laboratory, Huazhong Agricultural University, Wuhan, China; ^4^ Global Institute for Food Security, University of Saskatchewan, Saskatoon, SK, Canada; ^5^ Northeast Institute of Geography and Agroecology, Key Laboratory of Soybean Molecular Design Breeding, the Chinese Academy of Sciences, Harbin, China; ^6^ Department of Forestry Sciences, NEIKER-BRTA, Centro de Arkaute, Vitoria-Gasteiz, Spain; ^7^ Department of Biochemistry, Molecular Biology, Entomology and Plant Pathology, Mississippi State University, Mississippi, MS, United States

**Keywords:** hormone crosstalk, abscisic acid, signaling, regeneration or growth, crop improvement

As a sesquiterpene phytohormone derived from epoxycarotenoid cleavage, abscisic acid (ABA) is extensively involved in the regulation of various plant growth processes as well as adaptive responses to biotic and abiotic stresses (Nambara and Marion-Poll, 2005; Hirayama and Shinozaki, 2007; Yoshida et al., 2014; Wang et al., 2020). In this special issue “*Roles and Regulatory Mechanisms of ABA in Plant Development*”, 4 articles were published that explore ABA involvement in different developmental processes, covering outstanding advances in the fundamental roles of ABA in diverse plant research fields.

Previously, important functions of ABA biosynthetic pathways have been deciphered through molecular‐genetic, biochemical, and pharmacological approaches (Koornneef et al., 1982; Giraudat et al., 1992; González-Guzmán et al., 2002). A core signaling module involving “PYR/PYL/RCAR-PP2C-SnRK2-AREB/ABF” has been proposed and experimentally validated to play key roles in plant ABA signaling (Ma et al., 2009; Cutler et al., 2010). In the ABA signaling pathway, ABA is first recognized by the receptor proteins PYRABACTIN RESISTANCE (PYR)/PYR1-LIKE (PYL)/REGULATORY COMPONENTS OF ABA RECEPTORS (RCAR), and then interact with the clade A protein phosphatases of type 2Cs (PP2Cs). PP2Cs inactivation releases the inhibition on Sucrose non-fermenting 1-related protein kinases 2 (SnRK2) protein kinases that phosphorylate downstream ABA-responsive element binding factors (ABFs) to modulate ABA responses (Fujii et al., 2009; Park et al., 2009; Chen et al., 2020).

As the core element in ABA signaling pathway, PYR/PYL/RCAR family has been reported to play essential roles (Park et al., 2009). A recent study by Shang et al. identified novel pathways to modify ABA signaling through PYR1. In Arabidopsis, Brassinosteroid-Insensitive1-Associated Receptor Kinase1 (BAK1) interacts with PYR1 and phosphorylates PYR1 at the T137 and S142 sites. Phosphorylated PYR1 mainly exists in a monomeric form and increases the degree of complex formation with ABI1 and the ABA binding capacity. New findings presented in this issue by Shang et al. demonstrated that in addition to BAK1 interaction with OST1, importantly BAK1 also positively modulates ABA signaling through interaction with PYR1. This research further expanded our knowledge of the ABA signaling pathway.

The plant cell wall is structurally complex and pectin is a major component of it (Klis et al., 2006). When plant cells encounter stress, cell wall key components, such as pectin, pectin methylesterase (PME), and apoplastic Ca^2+^ will be reconstructed (Hamann et al., 2009; Wu et al., 2010). Wu et al. identified and characterized an Arabidopsis type-II *PME* gene *PME53*, which encodes a cell wall deposited protein and is highly expressed in guard cells as an abscisic acid (ABA)-regulated gene. They found that *PME53* regulates the activity of the core stomatal transcription factors SCRM and MUTE to modulate the development of stomatal and the flexibility of guard cell wall, thereby enhancing the adaptation of Arabidopsis to temperature changes. This research provided a novel perspective on ABA-mediated adaptive response of plant cell development to environmental stress.

In addition to key functions in stress response, ABA is also involved in several other important physiological processes in plants including seed maturation. Gupta et al. reviewed that ABA plays a key role in fruit development and ripening. In the later fruit ripening stage, the export of phloem ABA was decreased significantly, and ABA accumulation occurred. The interaction of ABA with ethylene and other plant hormones may play an essential role in fruit growth and ripening.

Plants also use complex signaling systems regulated by light and abscisic acid (ABA) components to optimize growth and development in different situations. Plants might reduce CO_2_ entry into leaves and limit photosynthesis by controlling stomatal aperture in response to stress conditions (Dong et al., 2015; Kuromori et al., 2018; Yoshida et al., 2019). However, it is still a mystery that how ABA and light signals are integrated at the molecular level? ABI5 (Abscisic acid-insensitive 5) is a key signaling hub in the ABA pathway. When plants sense the ABA signal, SnRK2 protein kinases activate the ABI5 subfamily proteins to promote the expression of ABA-responsive genes (Furihata et al., 2006; Umezawa et al., 2009). Recent in silico analysis of the public datasets performed by Bulgakov and Koren found that ABI5 is a main player that links ABA and light signaling during plant development. It is important to understand the interactions of ABA and light signals to improve the photosynthetic efficiency of crops, especially under climate challenged growth conditions.

Altogether, the contributions published in this special issue captured latest excellent advances providing new insights into ABA regulation of plant growth and development. The new research findings reported by Shang et al. and Wu et al. revealed novel components or pathways involved in ABA signaling. Light is an essential signal that regulates the physiological cycle, basic photomorphogenetic pathways, and secondary metabolites of plants (Chen et al., 2004). However, the role of abscisic acid (ABA) signaling in light response is still poorly understood. Bulgakov and Koren and Gupta et al. systematically reviewed the network between ABA and light signals or other phytohormones in the regulation of plant developmental programs. Together, in view of these research advances, future prospects targeting ABA signaling pathway through different strategies to regulate plant growth and development especially under a range of environmental challenges are very promising. We wish to thank all the authors for their contributions and the reviewers for their critical assessments of these articles. We also thank the *Frontiers in Plant Science* for giving us the opportunity to serve as guest editors of the Research Topic “*Roles and Regulatory Mechanisms of ABA in Plant Development*”.

## Author contributions

All the authors participated in the editing of this Research Topic. GL wrote the draft, and all the other authors provided suggestive comments on the editorial. All authors contributed to the article and approved the submitted version.

## Funding

This research was funded by MINECO project (AGL2016-76143-C4-3R), MICINN (PID2020-112627RB-C32), CYTED (P117RT0522), DECO (Basque government) and MULTIFOREVER project, supported under the umbrella of ERA-NET Cofund ForestValue by ANR (FR), FNR (DE), MINCyT (AR), MINECO-AEI (ES), MMM (FI), VINNOVA (SE) [ForestValue has received funding from the European Union’s Horizon 2020 Research and Innovation Programme under grant agreement no. 773324].

## Conflict of interest

The authors declare that the research was conducted in the absence of any commercial or financial relationships that could be construed as a potential conflict of interest.

## Publisher’s note

All claims expressed in this article are solely those of the authors and do not necessarily represent those of their affiliated organizations, or those of the publisher, the editors and the reviewers. Any product that may be evaluated in this article, or claim that may be made by its manufacturer, is not guaranteed or endorsed by the publisher.
